# Evaluating the effects between metal mixtures and serum vaccine antibody concentrations in children: a prospective birth cohort study

**DOI:** 10.1186/s12940-020-00592-z

**Published:** 2020-04-10

**Authors:** Barrett M. Welch, Adam Branscum, G. John Geldhof, Sharia M. Ahmed, Perry Hystad, Ellen Smit, Sakila Afroz, Meghan Megowan, Mostofa Golam, Omar Sharif, Mahmuder Rahman, Quazi Quamruzzaman, David C. Christiani, Molly L. Kile

**Affiliations:** 1 College of Public Health and Human Sciences, Oregon State University, Milam Hall, Room 101, Corvallis, OR 97331 USA; 2 Oregon Health and Sciences University, Portland, OR USA; 3 Dhaka Community Hospital Trust, Dhaka, Bangladesh; 4 Department of Environmental Health, Harvard University, Boston, USA

**Keywords:** Metal mixture, Arsenic, Lead, Manganese, Humoral immunity, Pregnancy, Windows of susceptibility, Developmental immunotoxicity, Diphtheria, Tetanus

## Abstract

**Background:**

Many populations are exposed to arsenic, lead, and manganese. These metals influence immune function. We evaluated the association between exposure to single and multiple metals, including arsenic, lead, and manganese, to humoral immunity as measured by antibody concentrations to diphtheria and tetanus toxoid among vaccinated Bangladeshi children. Additionally, we examined if this association was potentially mediated by nutritional status.

**Methods:**

Antibody concentrations to diphtheria and tetanus were measured in children’s serum at age 5 (*n* = 502). Household drinking water was sampled to quantify arsenic (W-As) and manganese (W-Mn), whereas lead was measured in blood (B-Pb). Exposure samples were taken during pregnancy, toddlerhood, and early childhood. Multiple linear regression models (MLRs) with single or combined metal predictors were used to determine the association with antibody outcomes. MLR results were transformed to units of percent change in outcome per doubling of exposure to improve interpretability. Structural equation models (SEMs) were used to further assess exposure to metal mixtures. SEMs regressed a latent exposure variable (*Metals*), informed by all measured metal variables (W-As, W-Mn, and B-Pb), on a latent outcome variable (*Antibody*), informed by measured antibody variables (diphtheria and tetanus). Weight-for-age z-score (WFA) at age 5 was evaluated as a mediator.

**Results:**

Diphtheria antibody was negatively associated with W-As during pregnancy in MLR, but associations were attenuated after adjusting for W-Mn and B-Pb (− 2.9% change in diphtheria antibody per doubling in W-As, 95% confidence interval [CI]: − 7%, 1.5%). Conversely, pregnancy levels of B-Pb were positively associated with tetanus antibody, even after adjusting for W-As and W-Mn (13.3%, 95% CI: 1.7%, 26.3%). Overall, null associations were observed between W-Mn and antibody outcomes. Analysis by SEMs showed that the latent *Metals* mixture was significantly associated with the latent *Antibody* outcome (β = − 0.16, 95% CI: − 0.26, − 0.05), but the *Metals* variable was characterized by positive and negative loadings of W-As and B-Pb, respectively. Sex-stratified MLR and SEM analyses showed W-As and B-Pb associations were exclusive to females. Mediation by WFA was null, indicating *Metals* only had direct effects on *Antibody*.

**Conclusions:**

We observed significant modulation of vaccine antibody concentrations among children with pregnancy and early life exposures to drinking water arsenic and blood lead. We found distinct differences by child sex, as only females were susceptible to metal-related modulations in antibody levels. Weight-for-age, a nutritional status proxy, did not mediate the association between the metal mixture and vaccine antibody.

## Background

Globally, arsenic, manganese, and lead are commonly encountered metals that can negatively impact public health. While many studies have focused on examining the reproductive and neurotoxic effects of these metal exposures on children’s health [1–4], few have examined their impact on immune functioning. Yet there is experimental and epidemiologic evidence that arsenic, manganese, and lead can affect the immune system. Experimental studies also show that arsenic impairs the function of T-cells, macrophages, and several other immune system cells [5–7]. Prospective cohort studies report that arsenic exposure increases infectious disease risk in the first year of life [8]. Additionally, prenatal and early childhood arsenic exposure is associated with suppressed cell-mediated immunity and modulation of humoral immunity via altered immunoglobulin (Ig) profiles [9–12]. Manganese plays an important role in nutritional immunity as it is sequestered cellularly to respond to infection [13]. However, there is evidence that high levels of manganese may impair immune function. Experimental studies in rodents and birds have shown that manganese exposure can cause a significant changes in lymphocyte responses and reduced antibody production [14], as well as increased susceptibility to bacterial and viral infection [15, 16]. Although epidemiologic evidence of manganese-related immunological effects is primarily restricted to occupational studies of adult men, high occupational exposure has been associated with significantly altered lymphocyte profiles (e.g. reduced total T-cells and serum Ig) [17, 18]. Lead exposure has had a long-standing concern over its capacity to influence immune function [19]. Experimental studies show elevated lead exposure causes increased susceptibility to bacterial and viral challenge in rodents [20–22], along with depressed humoral immune function following chronic low-level exposure [23]. Although lead has been shown to target both macrophages and T-cells, the influence on B-cell activity is unclear [24]. Epidemiologic evidence has shown that lead exposure is associated with increases in total serum IgE (a type-I hypersensitivity precursor), inflammatory cytokine profiles (e.g. increased Th2:Th1 ratio), and asthma severity or atopy [25–28]. Early life lead exposure in children has also been associated with reduced humoral immunity and vaccine response [27, 29, 30].

Here, we aim to investigate the association of humoral immunity and exposure to arsenic, manganese, and lead in children using a prospective birth cohort recruited in Bangladesh where children are frequently exposed to elevated environmental exposures to all three metals [31–33]. Due to previous public health efforts to reduce waterborne disease in the 1970’s, the population of Bangladesh switched its source of drinking water from surface water to groundwater which unfortunately contains high levels of arsenic [34]. Although mitigation efforts have helped to decrease arsenic exposures, recent surveys show that over 12% of household tubewells still contain drinking water arsenic concentrations (W-As) that exceed the Bangladeshi standard of 50 μg/L, and at least twice that number drink water above the 10 μg/L World Health Organization (WHO) guideline [35]. The groundwater in Bangladesh also has high levels of manganese [36]. Approximately 40% of household tubewells in the country are estimated to contain drinking water manganese concentrations (W-Mn) that exceed the former WHO health-based guideline of 400 μg/L [31, 36]. Furthermore, nearly 60% of tubewells in Bangladesh exceed health-based guidelines for either W-As or W-Mn [36]. There is also evidence of widespread elevated blood lead levels (B-Pb) in both urban and rural Bangladeshi children [3, 37]. The potential sources of children’s lead exposure in Bangladesh include air pollution, industrial discharges, glazed ceramic dishware, paint, contaminated food and medicine [38].

In the past two decades Bangladesh has made significant advances in reducing the burden of childhood infectious disease and mortality, which has largely been attributed to increased vaccination coverage, improved surveillance, expanded capacity building programs, and micronutrient supplementation initiatives [39, 40]. Since the vaccination coverage is very high among this population, we aimed to examine the single and combined exposure to arsenic, manganese, and lead on vaccine antibody concentrations which provide a feasible and clinically relevant biomarker of humoral immunity and potential developmental immunotoxicity [41, 42]. We hypothesized that higher concentrations of W-As, W-Mn, and B-Pb would be associated with lower diphtheria and tetanus antibody levels. Additionally, we hypothesized that nutritional status of children at age 5 would mediate the association between metal exposures and vaccine antibody. We complement standard multiple regression techniques with structural equation modeling (SEM) to assess how combined metal exposures may influence humoral immunity.

## Methods

### Study population

The study population was part of a prospective birth cohort recruited in the Munshiganj and Pabna districts of Bangladesh that has been described in detail elsewhere [3, 10, 43]. Briefly, pregnant women were recruited to examine associations between chronic arsenic exposure and pregnancy-related outcomes [43]. Women were required to have an ultrasound confirmed singleton pregnancy of ≤16 weeks gestation, use a tubewell for their primary drinking water source, and agree to utilize prenatal care services provided by Dhaka Community Hospital Trust [43]. Participating families were re-contacted twice for follow up. The first follow up visit occurred when children were aged 12 months and/or at 20–40 months to investigate the effects of metals and neurodevelopment [3]. The second follow up visit occurred when the children were aged 4–5 years old to investigate the effect of arsenic and other metals on immune functioning. Details of cohort follow-up procedures and loss to follow-up have been described in detail elsewhere [10].

Briefly, children were eligible for this study if they provided a blood draw at 5 years of age (± 2 months) and had received all three doses of the government-provided Hib pentavalent vaccine confirmed by government-provided vaccination records. A total of 502 children provided blood samples that were analyzed for serum antibodies. Study protocols were approved by the human research committees at Oregon State University, Harvard School of Public Health, and Dhaka Community Hospital Trust. Informed consent was obtained from each family and child prior to data collection.

### Environmental and biomarker metal measurements

Arsenic and manganese concentrations were measured in household drinking water (W-As and W-Mn, respectively). Water samples were collected from the tubewell that each family indicated was their primary source of drinking water at each study visit. Water sample collection and analysis was consistent between study visits. Briefly, field collection included purging the tubewell for 1-min before collecting water samples in 50-ml polyethylene tubes and then acidifying with ultrapure nitric acid. Samples were maintained at room temperature preceding analysis by inductively-coupled plasma mass spectrometry following US EPA method 200.8. Our method limit of detection (LOD) for W-As and W-Mn was 0.5 μg/L. W-As was below the LOD for 16 (3.2%), 78 (16.5%), and 157 (31.3%) samples measured during pregnancy, toddlerhood, and early childhood, respectively. A single value of W-Mn was below the LOD measured during early childhood. Final concentrations of W-As and W-Mn were calculated consistently to our previous study of W-As [10], whereby measurements taken during consecutive study visits were averaged to represent pregnancy, toddlerhood, and early childhood study periods. Between each pair of consecutive study visits, a child was assigned the average value if concentrations were available, or the single non-missing value if only one time period was available.

Lead (B-Pb) was measured in blood. Single measurements of B-Pb were collected at birth in cord blood, and fingerstick lead tests were conducted when children were age 20–40 months (B-Pb_pregnancy_ and B-Pb_toddlerhood_), and between ages 4–5 years (B-Pb_childhood_). Sample collection and laboratory procedures have been previously described in detail [2]. Briefly, umbilical cord venous blood was collected in trace element-free tubes at the time of delivery. Samples were kept at 4 °C and shipped to Harvard T.H. Chan School of Public Health for trace metal analysis. Lead concentrations were measured using a dynamic reaction cell-inductively coupled plasma mass spectrometer. Postnatal lead concentrations were measured in whole blood when the child was age 20 to 40 months (B-Pb_toddlerhood_) and again when the child was 4–5 years (B-Pb_childhood_). B-Pb_toddlerhood_ and B-Pb_childhood_ samples were measured using portable LeadCare II instruments (Magellan Diagnostics, Billerica, MA, USA) that have a reportable range of 3.3–65 μg/dL.

Out of 502 participants, concentrations of W-As and W-Mn were missing for 1 (0.2%), 29 (5.8%), and 0 children at pregnancy, toddlerhood, and early childhood, respectively. Concentrations of B-Pb were missing for 3 (0.6%), 172 (34.3%), and 6 (1.2%) children at pregnancy, toddlerhood, and early childhood, respectively.

### Vaccine antibody measurements

Infants from participating families were vaccinated according to the official Bangladesh vaccination program which includes administration of the Hib pentavalent vaccine at ages 6 weeks, 10 weeks, and 14 weeks [44]. This vaccine contains antigens to elicit protection for diphtheria and tetanus toxoid. Diphtheria and tetanus toxoids are both classic protein antigens that rely on T-helper cells for primary and recall antibody-mediated responses [45, 46]. Blood samples provided by children were used to quantify concentrations of diphtheria and tetanus antibody as described in detail elsewhere [10]. Briefly, 2 mL of venous blood was collected in vacutainers and allowed to clot at room temperature. Blood was promptly centrifuged to collect serum and then stored at − 20 °C until transported on dry ice to Oregon State University to be stored at − 80 °C. Diphtheria and tetanus antibody were measured by enzyme-linked immunosorbent assays using antibody-specific test kits following manufacturer protocols (Institut Virion-Serion GmbH, Würzburg, Germany).

### Description of covariates

Variables considered relevant to all three metal exposure variables and antibody outcomes were considered to be potential confounders. This included self-reported maternal education (illiterate or minimal writing, primary, and ≥ secondary), breastfeeding duration (months), and child sex (male, female). These variables were chosen a priori as potential confounders based on their known association with concentrations of arsenic, manganese, and lead exposures and vaccine-antibodies [47–49]. The weight measurements of children measured at age 5 years were converted to z-scores based on child age and sex using the WHO Child Growth Standards. Macro packages specific for children age ≤ 60 months (WHO Anthro, version 3.2.2) and > 60 months (WHO AnthroPlus, WHO Reference 2007) were used to calculate weight-for-age z-scores (WFA).

### Statistical analysis

#### Multiple linear regression

Multiple linear regression models (MLRs) were used to evaluate the association between concentrations of vaccine-specific antibodies and the three types of metals. Antibody and metal variables were log_2_-transformed to improve the interpretation of regression coefficients. Because both metals concentrations and antibody outcomes were log_2_-transformed, we report MLR results as the percent change (%) in median antibody concentration for a doubling of the given exposure (i.e. (2^x^ – 1) × 100%, where x represents individual coefficient values). Missing values of metal predictors were imputed for any metal exposure variables with > 1 observation missing, which included W-As_toddlerhood_, W-Mn_toddlerhood_, B-Pb_pregnancy_, B-Pb_toddlerhood_, and B-Pb_childhood_. Missing values were imputed using multiple imputation in chained equations (MICE) with predictive mean matching (PMM) in variable-specific univariate chained eqs. A total of 20 datasets were generated by MICE for each imputed variable. Of the 502 children with complete antibody measurements, 496 (99%) had complete data available after multiple imputation of exposure variables, while 6 children (1%) were excluded because of missing covariate data. Non-imputed MLR analyses were conducted as a sensitivity analysis (Additional file 1, Tables S4-S6).

Initial analyses included separate MLRs with age-5 antibody concentration as the dependent variable and arsenic, manganese, or lead measurements as the predictors. First, individual models were used to assess the associations between arsenic, manganese, or lead exposure variables at all three age periods on antibody-specific concentrations (i.e. single model included pregnancy, toddlerhood, and childhood exposure variables of one metal). This *single element* model provided a relative comparison of age-specific associations within a single type of metal exposure. Then, a *combined* model was used to assess antibody-specific associations to all metals simultaneously (i.e. all 9 exposure variables included in same model). This model offered the same comparison as the previous model, but also provided a relative comparison of associations between metals. Effect modification by child sex was assessed by stratifying models by sex. Additionally, to evaluate the potential moderating effect of differences by clinic site, separate models were run stratified by clinic location (i.e. Pabna or Munshiganj).

All adjusted models included maternal education, breastfeeding duration, and child sex (excluded in sex-stratified models). Assumptions of normality and variance homogeneity were visually examined and empirically evaluated by descriptive statistics. The statistical significance level was set at α = .05. Multivariable regression analyses were performed using Stata version 14.2 (StataCorp LP, College Station, TX).

#### Structural equation modeling

Structural equation models (SEMs) were developed to further characterize the association of antibody concentrations and combined metals exposures. SEMs are an analytic tool for examining the relationships between observed (measured) and latent (non-measured) variables, which provides an approach to evaluate how well data correspond to a theoretical framework or hypothesis [50]. Compared to MLR, SEMs can account for measurement error of exposures and outcomes [51]. Additionally, because data from multiple exposures and multiple outcomes are considered simultaneously, SEMs reduce issues of multiple comparisons and provide greater statistical power by pooling variables [51, 52]. The latent variable represents the shared variance between the observed variables (indicators), while the residual errors of each observed variable represent the unexplained variance (assumed to represent random error). In SEM diagrams latent variables are represented by ovals while observed variables are represented by rectangles. Single headed arrows represent regressions and double-headed arrows indicate covariance. Factor loadings (λ) refer to the single headed arrows between the latent variable and the observed variables it informs, while single-headed arrows between the latent variable and an outcome variable refer to standard beta coefficients (β).

In this analysis, our a priori SEM hypothesized a latent exposure variable referred to as *Metals* was a regression predictor of a latent antibody outcome variable referred to as *Antibody* (Fig. 2). The *Metals* variable was informed by the concentrations of W-As, W-Mn, and B-Pb at all three time periods (pregnancy, toddlerhood, and childhood), while the *Antibody* variable was informed by concentrations of diphtheria and tetanus antibody. This model implies that the latent *Metals* variable represents the combined metals exposure, whereby all markers of early life metal exposures were considered error-prone indicators of the same latent exposure. Similarly, the latent *Antibody* variable is implied to represent the combined vaccine antibody outcomes, as indicated by diphtheria and tetanus antibody. The *Metals* and *Antibody* variables were indicated by observed variables but were themselves unmeasured and thus have no inherent metric. Therefore, to set a measurement scale we implemented latent variable standardization by fixing latent variances to 1.0 and latent means/intercepts to 0 [50]. This standardization allowed us to 1) determine the association of the various metal indicators with the latent *Metals* variable despite different measurement scales (i.e. μg/L in water versus μg/dL in blood) and 2) determine the association of *Metals* with *Antibody* despite no inherent latent variable metric. The standardized factor loadings allowed comparison of the relative influence among variables and can be interpreted similarly to beta coefficients calculated in standard linear regression analyses.

Maximum likelihood estimation with robust standard errors was used to fit SEMs. Incomplete observations of exposure variables were assumed to have information missing at random, thus allowing imputation based on the full information maximum likelihood [53]. Global model fit was evaluated using the following indices: Comparative Fit Index (CFI), Tucker-Lewis Index (TLI), and Root Mean Square Error of Approximation (RMSEA). Table S1 in Additional file 1 provides the criteria used to determine goodness of fit, with all SEMs required to meet good or acceptable fit for each model fit index. Measurement error was determined a priori to be based on within-metal observed variables, whereby residual errors for repeated measures of each type of exposure were correlated (e.g. all W-As measurements correlated with each other). Modification indices were used to verify that specified error correlations were improving model fit using likelihood ratio tests (LRT) between 1) a null model with no specified correlations between residual errors and 2) a model specifying residual errors correlations. Improved model fit was indicated by a LRT with *p*-value< 0.05 [50]. Consistent with our multiple regression analyses, covariates in adjusted analyses included maternal education, breastfeeding duration, and child sex (not included in sex-stratified models).

To determine if nutritional status mediated the association between *Metals* and *Antibody*, the previous a priori SEM was expanded to consider WFA, an indicator of nutritional status, as a mediator (Fig. 3). It should be noted that in this mediation analysis the term “effect” refers to statistical and not causal effect. The direct effect was the direct association between *Metals* and *Antibody*. The indirect effect was considered as the product of the associations (i.e. regression coefficients) between *Metals* and WFA and between WFA and *Antibody*. The total effect was the sum of the direct and indirect effects. Statistical significance of SEM estimates was evaluated with 95% confidence intervals and an α = 0.05. SEM analyses were performed in R (R Version 3.4.2) using the “Lavaan” package [54].

## Results

**Fig. 1:**
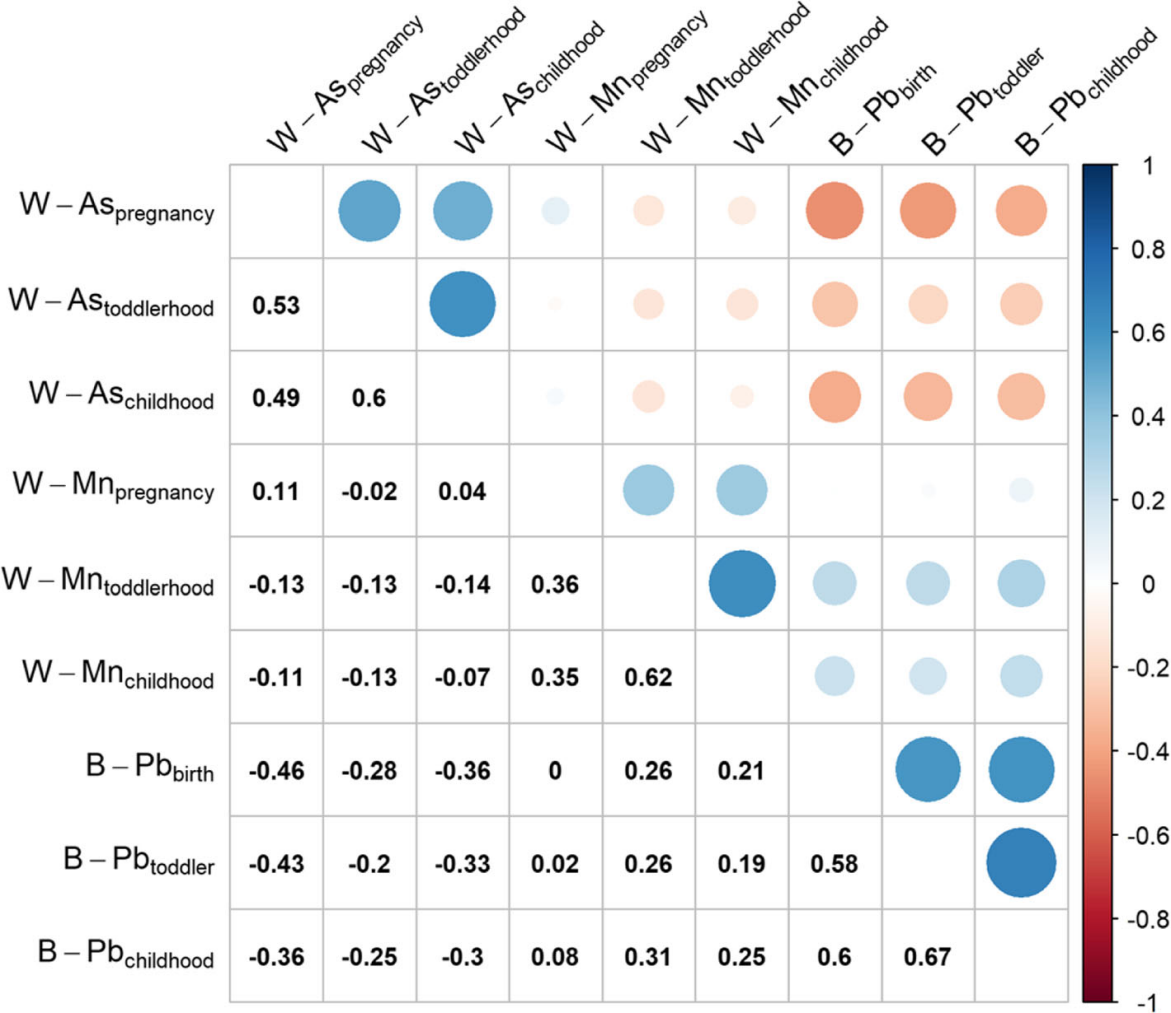
Matrix of spearman correlation coefficients (ρ) for concentrations (log_2_-transformed) of drinking water arsenic (W-As), manganese (W-As), and blood lead (B-Pb) among children with antibody outcomes during pregnancy, toddlerhood, and childhood. Blue and red indicate positive and negative correlation, respectively, while the size of the circle corresponds to magnitude of the correlation coefficient

The characteristics of participants contributing serum samples at age 5 years are presented in Table 1. Most mothers had a secondary education and breastfed for at least 2 years. Girls tended to have smaller stature as indicated by lower age 5 WFA values than boys (*p* = .005). Serum antibody concentrations were higher for tetanus than for diphtheria antibody. The highest median concentrations of W-As and B-Pb were observed during toddlerhood. Median B-Pb reached or exceeded recommended health-based guideline limits (i.e. > 5 μg/dL) during the toddlerhood and childhood periods, while median W-Mn exceeded health-based guidelines (i.e. > 400 μg/L) during all three age periods. Distributions of metal and antibody variables did not significantly differ by child sex. The within-metal concentrations between age periods were positively correlated (0.35 ≤ ρ ≤ 0.67), with the strongest correlations observed between the toddlerhood and childhood age periods (Fig. 1). The correlations between prospective metal measurements were more variable as W-As was negatively correlated with W-Mn and B-Pb, while W-Mn was positively correlated with B-Pb.

**Table 1:** Characteristics of children in Bangladeshi prospective cohort who provided serum antibody samples at age 5 years

Variable		Child sex		
	Overall	Males	Females	
	*N* = 502	(*n* = 255)	(*n* = 246)	*p-value*
Maternal Education, *n* (%)				
illiterate	52 (10.4%)	29 (11.4%)	23 (9.3%)	0.76
primary	187 (37.3%)	94 (36.9%)	93 (37.8%)	
≥ secondary	262 (52.2%)	132 (51.8%)	130 (52.8%)	
Total duration of breastfeeding, median (IQR), months	24.0 (19.0, 36.0)	24.0 (18.0, 36.0)	24.0 (24.0, 36.0)	0.41
WFA	−1.3 (−2.0, − 0.6)	− 1.2 (− 1.9, − 0.6)	− 1.5 (− 2.1, − 0.8)	0.005
Antibody concentration at age 5, median (IQR), IU/mL				
Diphtheria	0.06 (0.03, 0.12)	0.06 (0.03, 0.13)	0.06 (0.03, 0.12)	0.78
Tetanus	0.21 (0.09, 0.40)	0.22 (0.10, 0.42)	0.20 (0.09, 0.37)	0.12
Arsenic (W-As), median (IQR), μg/L				
pregnancy	3.2 (1.2, 23.7)	3.2 (1.2, 24.2)	3.3 (1.2, 22.3)	0.70
toddlerhood	7.1 (0.8, 47.7)	7.9 (0.9, 47.7)	6.9 (0.7, 49.4)	0.41
childhood	2.7 (0.4, 38.4)	3.6 (0.4, 37.2)	2.5 (0.4, 39.1)	0.31
Manganese (W-Mn), median (IQR), μg/L				
pregnancy	565.0 (344.8, 796.0)	565.0 (350.5, 789.0)	569.5 (340.0, 814.0)	0.62
toddlerhood	741.0 (322.5, 1478.5)	776.5 (358.0, 1560.0)	710.0 (308.0, 1410.0)	0.38
childhood	748.6 (304.3, 1483.7)	721.3 (322.1, 1512.0)	806.5 (252.6, 1422.6)	0.91
Lead (B-Pb), median (IQR), μg/dL				
pregnancy	3.1 (1.6, 5.6)	3.2 (1.5, 5.2)	3.0 (1.6, 5.8)	0.78
toddlerhood	6.4 (4.3, 10.0)	6.5 (4.4, 9.5)	6.0 (4.0, 10.4)	0.77
childhood	4.7 (2.3, 7.0)	5.0 (2.9, 7.9)	4.6 (2.3, 6.5)	0.05

Note: *p*-values Pearson chi-squared or Kruskal-Wallis rank sum differences in distribution of variable between sexes; *IQR* interquartile range (25th, 75th percentiles); *IU* international units; *W*- drinking water; *WFA* weight-for-age z-score

MLR analyses between metals and antibody concentrations are presented in Table 2. Overall, higher W-As_pregnancy_ was associated with lower concentrations of diphtheria antibody (− 3.4% change per doubling in W-As, 95% CI: − 7.2, 0.6%). This association was slightly attenuated after adjusting for W-Mn and B-Pb (− 2.9, 95% CI: − 7.0, 1.5%). Whereas, higher B-Pb_pregnancy_ was associated with higher concentrations of tetanus antibody (10.2, − 0.6%, 22.1%), which showed stronger effects after adjusting for W-As and W-Mn (13.3, 95% CI: 1.7, 26.2%). No association was observed between W-Mn and either antibody outcome. MLR stratified by child sex showed that associations among girls likely drove the observed associations in the overall population (Additional file 1, Table S2-S3). Among girls, there was a negative association between diphtheria antibody and W-As_pregnancy_ that was attenuated after adjusting for B-Pb and W-Mn (− 5.8, 95% CI: − 11.2, − 0.2%), while there was a strong positive association between tetanus antibody and B-Pb_pregnancy_ (23.6, 95% CI: 7.0, 42.9%) in the combined metal model (Additional file 1, Table S2). Null associations were observed among boys regardless of the exposure variable or antibody type (Additional file 1, Table S3). Sensitivity analyses of MLR showed that multiple imputation procedures for missing exposure values did not influence overall study conclusions (Additional File 1, Table S4-S6).

**Table 2:** Results from adjusted multiple linear regression models of diphtheria and tetanus antibody outcomes for single and combined metal exposures among the overall study population (*n* = 502)

	Water arsenic		Water manganese		Blood lead	
	Change (%) (95% CI)		Change (%) (95% CI)		Change (%) (95% CI)	
	Single element^a^	Combined^b^	Single element^a^	Combined^b^	Single element^a^	Combined^b^
Diphtheria IgG antibody						
Pregnancy	−3.4 (−7.2, 0.6)	−2.9 (− 7.0, 1.5)	−1.6 (−8.3, 5.5)	−1.1 (− 7.9, 6.2)	4.7 (− 4.9, 15.3)	4.1 (− 5.9, 15.2)
Toddlerhood	1 (− 2.7, 4.9)	0.8 (− 2.9, 4.8)	2.7 (− 3.1, 8.8)	2.7 (− 3.2, 8.9)	5.9 (− 10.4, 25.2)	4.6 (− 11.9, 24.1)
Early childhood	1.7 (− 1.6, 5.1)	2 (− 1.4, 5.5)	− 0.7 (− 5.8, 4.8)	−1.1 (− 6.4, 4.5)	−6.4 (− 20.4, 10.1)	−6.2 (− 20.5, 10.5)
Tetanus IgG antibody						
Pregnancy	0.5 (− 3.8, 5.0)	2.6 (− 2.0, 7.5)	5.1 (− 2.5, 13.3)	5.4 (−2.3, 13.6)	10.2 (− 0.6, 22.1)	13.3 (1.7, 26.2)
Toddlerhood	− 0.8 (− 4.7, 3.3)	−1 (− 4.9, 3.1)	− 2.7 (− 8.5, 3.5)	−3.9 (− 9.7, 2.3)	1.6 (− 15.5, 22.2)	3.2 (− 14.1, 24.1)
Early childhood	−0.1 (− 3.6, 3.6)	0.4 (− 3.2, 4.1)	− 0.6 (− 6.1, 5.2)	−1.5 (− 7.1, 4.4)	2 (− 14.3, 21.4)	3.4 (− 13.2, 23.2)

Note: Missing values of arsenic, manganese, and lead were estimated by multiple imputation. All models are adjusted for maternal education, breastfeeding duration, and child sex. Outcomes are interpreted as percent change in median antibody concentration per doubling in given exposure

^a^ Models include includes single metal exposure category (arsenic, manganese, or lead) at all three periods of exposure

^b^ Model includes all metal exposure categories (arsenic, manganese, and lead) at all three periods of exposure

**Fig. 2:**
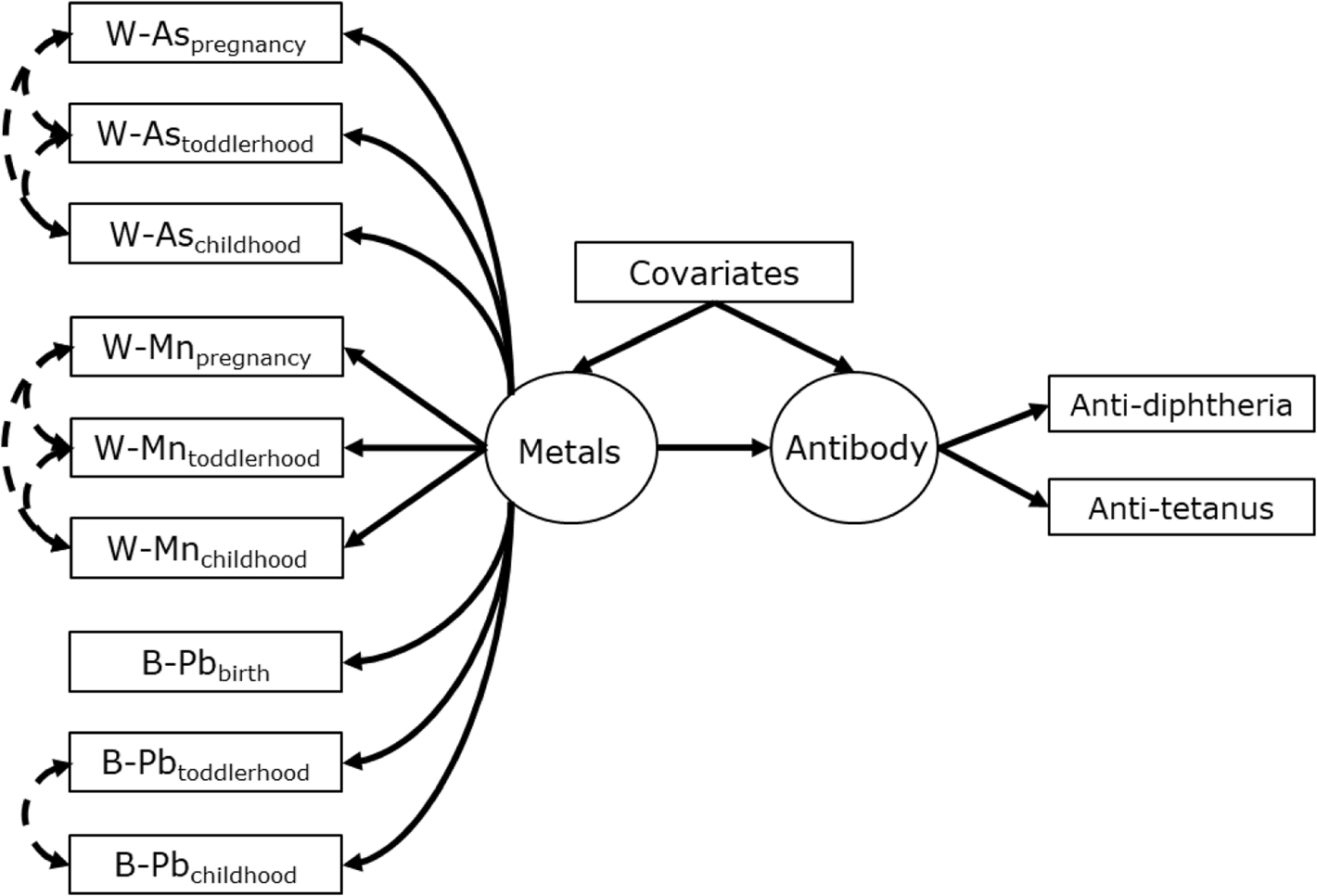
Structural equation model for the association between latent *Metals* exposure (left circle) informed by concentrations of W-As, W-Mn, and B-Pb at pregnancy, toddlerhood, and childhood (left rectangles) to latent *Antibody* concentration (right circle) informed by age-5 diphtheria and tetanus antibody (right rectangles). Note: Covariates (middle rectangle) include maternal education, child sex, and breastfeeding duration. Single headed arrows represent regressions between variables (factor loadings when observed variable is regressed on its latent construct). The double-headed dashed arrows represent specified correlations between residuals of observed variables

The SEM analysis first assessed the association between the hypothesized latent exposure and outcome variables, M*etals* and *Antibody*, as shown in Fig. 2. In the overall population (Table 3), a significant association was observed between the *Metals* exposure and *Antibody* outcome after covariate adjustment (β = − 0.14, 95% CI: − 0.25, − 0.03). However, the sex-stratified SEMs showed that the association was only observed among girls (β = − 0.31, 95% CI: − 0.45, − 0.17) as it was null among boys (adjusted β = 0.02, 95% CI: − 0.14, 0.17). Importantly, the standardized factor loadings (λ) of the observed variables indicating *Metals* displayed directional heterogeneity, meaning W-As loadings were positive while B-Pb and W-Mn loadings were negative (Table 4). This indicates that as W-As increased there were associated decreases in W-Mn and B-Pb within the latent *Metals* variable, which is consistent with the correlations between exposure variables we observed. Additionally, these loadings imply that the significant association between *Metals* and *Antibody* was negative with increases in W-As but positive with increases in B-Pb and W-Mn. The strongest loading onto *Metals* was observed for B-Pb_pregnancy_ (λ = − 0.81, 95% CI: − 0.88, − 0.75), while W-As_pregnancy_ had the strongest loading among W-As variables (λ = 0.59, 95% CI: 0.52, 0.66). The weakest loadings were observed for W-Mn variables, and W-Mn_pregnancy_ was the only exposure variable that did not significantly load on *Metals* (λ = 0.01, 95% CI: − 0.1, 0.1). The factor loadings for girls and boys were similar to those in the overall population, which demonstrated that the latent *Metals* represented the same underlying exposure process between sexes (i.e. any difference in *Antibody* to *Metal* association between sexes was not due to differences in exposure profiles).

**Table 3:** Results for SEM of latent *Metals* regressed on latent *Antibody* as shown in Fig. 2

Associations	Overall (*n* = 498)		Girls (*n* = 246)		Boys (*n* = 255)	
	Latent antibody β (95% CI)	*p-value*	Latent antibody β (95% CI)	*p-value*	Latent antibody β (95% CI)	*p-value*
Latent metal mixture						
Unadjusted	−0.16 (− 0.26, − 0.05)	0.003	−0.26 (− 0.4, − 0.131)	<.001	−0.04 (− 0.2, 0.12)	0.62
Adjusted	−0.14 (− 0.25, − 0.03)	0.01	−0.31 (− 0.45, − 0.17)	<.001	0.02 (− 0.14, 0.17)	0.82

Note: Covariates in adjusted model include maternal education, breastfeeding duration, and child sex (overall model only)

Fit indices for all models: CFI: 0.96–0.99; TLI = 0.95–0.98; RMSEA: 0.04–0.05

**Table 4:** Standardized factor loadings (λ) of observed (measured) exposures (W-As, W-Mn, and B-Pb) on the latent *Metals* variable and of observed outcomes (diphtheria and tetanus) on the latent *Antibody* variable. Factor loadings are relevant to SEMs diagrammed in Fig. 2

Variables	Overall (*n* = 498)		Girls (*n* = 246)		Boys (*n* = 255)	
	λ (95% CI)	*p-value*	λ (95% CI)	*p-value*	λ (95% CI)	*p-value*
Latent *Metals*						
Arsenic						
pregnancy	0.59 (0.52,0.66)	<.001	0.6 (0.5,0.7)	<.001	0.59 (0.5,0.69)	<.001
toddlerhood	0.34 (0.25,0.43)	<.001	0.32 (0.19,0.46)	<.001	0.35 (0.21,0.49)	<.001
childhood	0.43 (0.35,0.52)	<.001	0.36 (0.23,0.48)	<.001	0.5 (0.39,0.61)	<.001
Manganese						
pregnancy	0.01 (−0.1,0.11)	0.91	−0.08 (− 0.24,0.08)	0.34	0.05 (− 0.1,0.2)	0.53
toddlerhood	−0.24 (− 0.35,-0.14)	<.001	− 0.26 (− 0.41,-0.11)	0.001	−0.27 (− 0.41,-0.12)	<.001
childhood	−0.18 (− 0.27,-0.08)	<.001	−0.17 (− 0.3,-0.03)	0.02	−0.19 (− 0.33,-0.05)	0.01
Lead						
pregnancy	−0.81 (− 0.88,-0.75)	<.001	− 0.79 (− 0.88,-0.7)	<.001	−0.82 (− 0.92,-0.72)	<.001
toddlerhood	−0.69 (− 0.77,-0.61)	<.001	−0.72 (− 0.84,-0.6)	<.001	−0.67 (− 0.77,-0.56)	<.001
childhood	−0.71 (− 0.77,-0.64)	<.001	−0.81 (− 0.9,-0.71)	<.001	−0.63 (− 0.73,-0.53)	<.001
Latent *Antibody*						
Diphtheria	0.78 (0.71,0.85)	<.001	0.78 (0.71,0.85)	<.001	0.71 (0.65,0.77)	<.001
Tetanus	0.75 (0.62,0.89)	<.001	0.75 (0.62,0.89)	<.001	0.84 (0.68,1)	<.001

Note: Standardized loadings are restricted to values between 0 to 1.0, which are interpreted as correlations

**Fig. 3:**
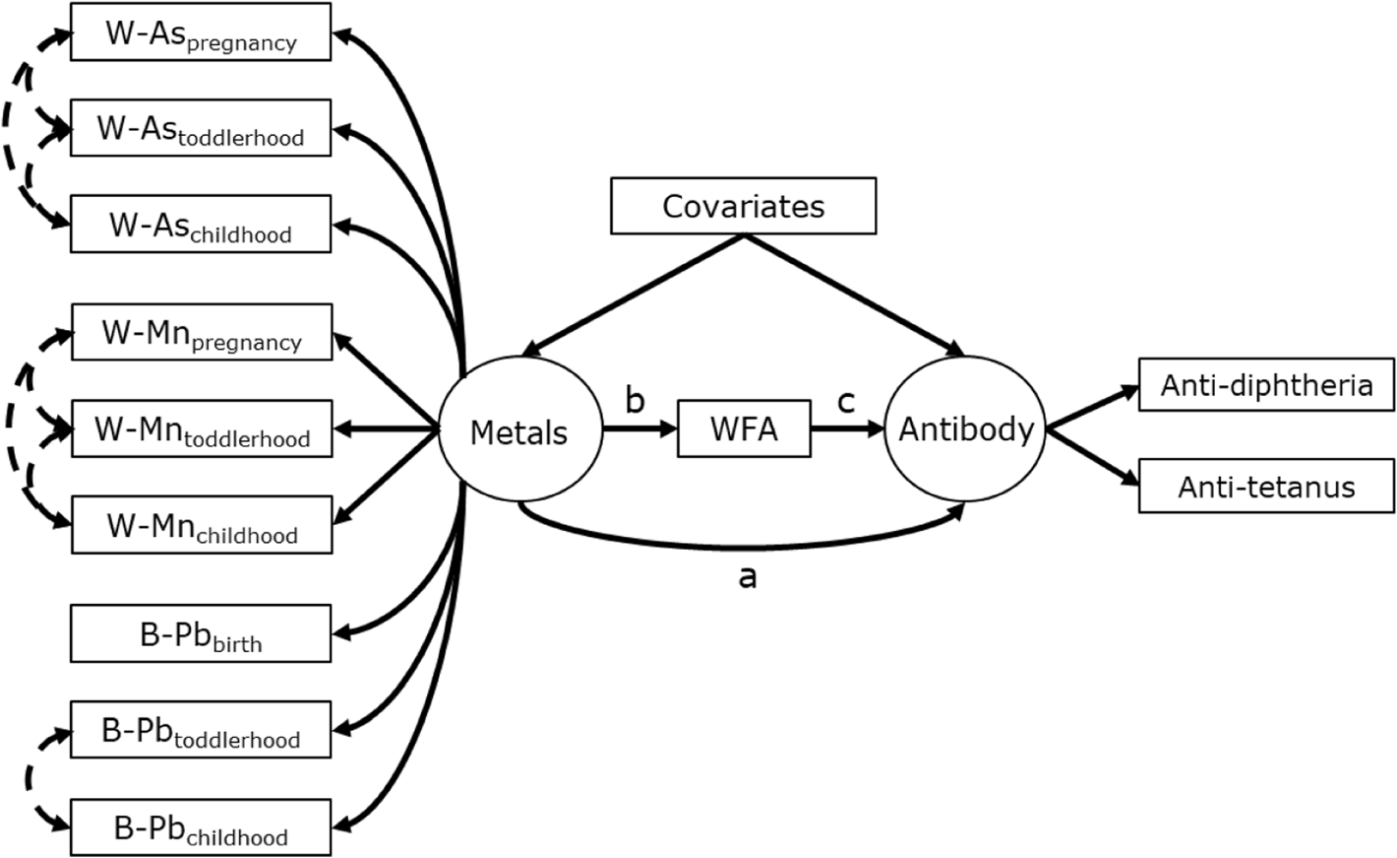
Hypothesized structural equation model for the association between latent *Metals* exposure and latent *Antibody* outcome concentrations that is mediated by weight-for-age (WFA). The potential pathways include the direct (a) and indirect (b × c) effects

To determine if the association observed between *Metals* and *Antibody* was mediated by nutritional status, WFA was evaluated as a mediator within the SEM (Fig. 3). Consistent with the previous model, the adjusted results showed a significant direct effect that was primarily driven by the association among girls (β = − 0.31, 95% CI: − 0.45, − 0.16, See Table 5). There was a significant association between WFA and *Metals* among girls (β = − 0.16, 95% CI: − 0.28, − 0.03), but not boys (β = − 0.07, 95% CI: − 0.21, 0.07). However, the indirect effect was null, as there was not a significant association between WFA and *Antibody,* regardless of child sex stratification. Thus, the total effect was similar to the direct effect in non-stratified and sex-stratified SEMs. The factor loadings of exposure indicators onto *Metals* were approximately equal to those from the previous model (data not shown). This signifies that the same negative associations between W-As and B-Pb were driving the estimated direct and indirect effects for the mediation SEM in Fig. 3 as discussed for the SEM in Fig. 2 (with and without WFA mediator).

**Table 5:** Results for SEM estimating the direct and indirect effects of latent *Metals* on latent *Antibody* with weight-for-age (WFA) mediation as shown in Fig. 3

	Overall (*n* = 498)		Girls (*n* = 246)		Boys (*n* = 255)	
	Latent antibody β (95% CI)	*p-value*	Latent antibody β (95% CI)	*p-value*	Latent antibody β (95% CI)	*p-value*
Latent metal associations						
*Direct effect*	−0.13 (− 0.25, − 0.02)	0.02	−0.31 (− 0.45, − 0.16)	<.001	0.02 (− 0.14, 0.18)	0.82
Indirect	−0.01 (− 0.02, 0.01)	0.29	−0.003 (− 0.03, 0.02)	0.76	−0.004 (− 0.02, 0.01)	0.53
WFA ~ metals	−0.11 (− 0.21, − 0.02)	0.02	−0.16 (− 0.28, − 0.03)	0.02	−0.07 (− 0.21, 0.07)	0.33
antibody ~ wfa	0.06 (−0.04, 0.16)	0.24	0.022 (−0.12, 0.16)	0.76	0.06 (−0.08, 0.2)	0.40
Total effect	−0.14 (− 0.25, − 0.03)	0.01	−0.31 (− 0.45, − 0.17)	0.76	0.02 (− 0.14, 0.17)	0.86

Note: Estimates are adjusted for the covariates indicating maternal education, breastfeeding duration, and child sex (overall model only)

Fit indices for all models: CFI: 0.95–0.97; TLI = 0.94–0.96; RMSEA: 0.03–0.04

## Discussion

In a large prospective birth cohort, we found metal exposure was both negatively and positively associated with vaccine antibody concentrations. Namely, W-As was associated with decreased antibody levels but B-Pb was positively associated with antibodies. Exposures that occurred during pregnancy had the greatest impact on these outcomes. We also observed effect modification by child sex, as girls were the most susceptible and drove the overall associations. Finally, SEM analyses provided additional evidence of the strong influence of B-Pb_pregnancy_ relative to other exposure variables, as it had the strongest (albeit negative) loading onto the latent *Metals* that likely influenced its significant association with latent *Antibody.* The positive association we observed for B-Pb was contrary to what we hypothesized. We originally hypothesized that lead exposure would be associated with signs of humoral immunosuppression based on previous evidence of lead-associated reductions in total IgG and vaccine-specific antibodies [26, 29, 30]. One cross-sectional study provided evidence of immunosuppression by observing a negative association between B-Pb and hepatitis B surface antibody titers among vaccinated elementary school children living near an e-waste facility [30]. However, associations were complicated by relatively high B-Pb in both the reference and exposed populations (B-Pb of 6.1 μg/dL vs 6.8 μg/dL, respectively), while the exposed population lived nearby an e-waste facility and was therefore likely exposed to other potentially immunotoxic chemicals that were not measured [30]. Additionally, other studies provide conflicting results by showing early life lead exposure has null [25, 55, 56], or positive [22, 57, 58], associations with total IgG antibody. Sarasua et al. (2000) conducted a large cross-sectional study (*n* = 2041) in the U.S. that found a positive association between B-Pb to total IgG, B-cell proportions, and T-cell proportions among children under age 3 years (*n* = 372, mean B-Pb = 7.0 μg/dL), but older age groups (3–6 years, 6–15 years, and 16–75 years) showed no significant associations between immune measures and B-Pb [57]. The study found that those children under age 3 in the moderate B-Pb range (5–15 μg/dL) had higher total IgG than those in the lower range (< 5 μg/dL), while other associations were driven by children in the highest B-Pb range (≥15 μg/dL) [57]. The weight of evidence seems to show that the suppressive effect of lead exposure on B-cells is weaker than T-cells [59, 60], which may partially explain why we did not observe negative associations.

Although our results are observational and should be interpreted cautiously, a potential biological explanation for the positive association we observed with B-Pb likely involves underlying lead-related shifts in the balance of T helper (Th) cell ratios. Studies of lead-induced immunomodulation in experimental and human populations have shown that elevated lead exposure reduces Th1 responses and promotes Th2 responses [24, 61–63]. Th1 cells have a primary function to activate certain immune cells like macrophages and stimulate CD8+ T-cells, while Th2 cells primarily function to increase antibody production via B-cell stimulation [64]. This alteration in Th cell ratios mostly occurs through the modulation of cytokine profiles that stimulate Th2 pathways (e.g. increased IL-2 and IL-4) and that reduce or inhibit Th1 pathways (e.g. decreased IFNγ and increased TGF-β) [61]. Although some IgG antibody production occurs through Th1 responses, the neutralizing antibody production, particularly IgE, is promoted more strongly by Th2 responses [64]. Further, lead exposure has been shown to enhance Th2 cytokines such as IL-4, IL-5, and IL-10 [63, 65–67], which help to drive the proliferation and differentiation of plasma cells to IgG antibody [64, 68]. Interestingly, there is some evidence suggesting that the amount of differential skewing between Th1 and Th2 responses following lead exposure may be dose-dependent, with lower exposure profiles (e.g. 4–10 μg/dL) potentially favoring Th1 dependent skewing [22, 69]. This may help explain the positive associations we observed, as our study population had relatively lower B-Pb exposure profiles corresponding to potential Th1 skewing. Additional studies should focus on describing the potential differential effects of low and high lead exposure on humoral immunity.

The negative associations we observed between W-As and vaccine antibody are consistent with our recent findings [10]. Experimental studies in various adult animal models have shown that arsenic exposure suppresses humoral immune function [6, 70]. Several large cross-sectional studies in the U.S. have provided evidence that suggests arsenic exposure increases the loss of protective antibodies to common viruses and anti-viral vaccines [71–73]. A case-control study nested within a prospective cohort in Bangladesh also observed that arsenic exposure potentially modulates humoral immunity regarding viral infection, as higher urinary arsenic was associated with increased susceptibility to hepatitis E viral infection among pregnant women [74]. In contrast to our results, multiple studies have observed positive associations between arsenic exposure and antibody profiles [11, 12, 75, 76]. A small cohort study in Bangladesh (*n* = 60) found that following booster vaccination, children living within a high W-As area had higher diphtheria and tetanus vaccine-related antibody concentrations, but lower blood counts, compared to children living in a low W-As area [12]. However, no pre-booster antibody titers were evaluated and antibody response was measured within a relatively short time frame following vaccination (21 days) [12], which prevents the results from being used to evaluate if arsenic affected long-term antibody waning. A larger study in a prospective birth cohort (*n* = 525) observed that first trimester maternal urinary arsenic was positively associated with total IgG measured at age 9 years [11]. There were also positive associations between urinary arsenic at all age periods (prenatal, 4.5 years, 9 years) and total IgG antibody among underweight children [11]. However, consistent with our findings, the authors observed that concentrations of mumps-specific IgG antibody following primary vaccination were negatively associated with childhood arsenic among children who that had the lowest pre-vaccination mumps antibody [11]. These results do suggest that although higher total IgG concentrations may be occurring within certain susceptible subgroups, potential impairment of humoral immunity can still be detected in the short-term period after primary vaccination.

Similar to previous studies that investigated the impact of arsenic or lead on humoral immunity in children [11, 26], we found a distinct difference in estimated associations by child sex. Previously, we demonstrated that girls, not boys, had significant negative associations between W-As_pregnancy_ to diphtheria and tetanus antibody [10]. Here, we have added weight to evidence of differential sex effects by showing that there were strong positive associations between B-Pb_pregnancy_ and tetanus antibody among girls, regardless of the modeling approach utilized. Other studies in children have also found sex-specific associations, but with the opposite sex or opposing directionality. Raqib et al. (2017) observed a positive association between urinary arsenic and total IgG among boys, but not girls [11], which contrasts with our negative association among girls. Contrary to our positive association among girls, Sun et al. (2003) observed a negative association between total IgG and B-Pb among girls [26]. Arsenic and lead have both been shown to modulate steroid sex hormone levels [77–80]. Sex hormones can strongly influence immune reactivity, with those of males generally considered immunostimulatory and those of females immunosuppressive [81, 82]. Therefore, developmental exposure to these metals could be modulating immunoglobulin levels via their effect on sex steroids. Better understanding of these potential biological mechanisms underlying immunomodulation by metals requires future studies in children to examine associations by sex and to determine any mediating roles of sex hormones.

Overall, manganese showed the weakest associations with vaccine antibody concentrations among the three metals. Manganese was not associated with either vaccine antibody in the overall population, while W-Mn_pregnancy_ was the only metal to not have a non-significant loading onto the latent *Metals* variable in SEM analyses. Our sex-stratified MLR analyses showed a potential positive association between W-Mn_pregnancy_ to tetanus antibody among girls, but this may be spurious as it was not observed elsewhere and we did not correct for multiple comparisons. The W-Mn concentrations were highly elevated in this population, as each age period had median concentrations exceeding 500 μg/L. The WHO discontinued its previous health-based guideline of 400 μg/L for W-Mn in 2011 based on the judgement that such exposure levels are uncommon [83]. However, results from our study and evidence from other regions in Bangladesh and numerous other countries demonstrate that, in fact, high exposure levels can be common in certain populations [84].. Because W-Mn is thought to be more bioavailable than other dietary sources of manganese among populations with rice-based diets such as Bangladesh [85], it is likely that the elevated W-Mn of women and children in this cohort represents an important source of manganese exposure. As manganese is an important metal involved with nutritional immunity, future studies should explore whether the nutrition of certain subpopulations (e.g. low socioeconomic groups, females) may be modulating the effect of excess manganese exposure on immune function.

Our results highlight that although arsenic and lead exposures may be influencing humoral immune status, especially within girls, they are likely doing so along different biological pathways. It is recognized that metals can have both inhibitory and stimulatory effects on the activation of various human lymphocytes [86]. Environmental exposures to arsenic and manganese have been independently associated with reductions in ratios of CD4+/CD8+ T-cell [18, 87, 88], which are signs of potential immunosuppression [89]. However, this reduced ratio has not been observed as consistently with regards to environmental lead exposure [59]. It is still difficult to determine whether the opposing associations observed with arsenic and lead are producing antagonistic or synergistic effects on humoral immunity. Future experimental and human studies could help address this knowledge gap by evaluating the differential effects of high to moderate exposure to each of these chemicals. Humoral immune function seems to be less prone to impairment by either arsenic or lead compared to cell-mediated immunity [8, 59]. Additional work examining the impact of both humoral and cell-mediated immunity in the context of metal mixtures would help determine the interplay between these interrelated immune processes and prevalent exposures.

We did not observe significant mediation by children’s growth scores for weight at age 5. Recently, we found that W-As_pregnancy_ had strong negative associations with vaccine antibody among children with potentially poorer nutritional status, as indicated by age 5 stunting or underweight [10]. Similarly, another prospective study in Bangladesh showed children with these indicators of poorer nutritional status had modulated humoral immunity following early life arsenic exposure [11]. Although these studies provide evidence that nutritional status likely moderates the effect of metals, our present results do not support the hypothesis that nutritional status is on the causal pathway between metals exposure and modulated humoral immunity. While WFA is considered a useful indicator of nutritional status, more comprehensive nutritional measurements (e.g. specific dietary surveys) of children would provide greater clarity on the potential influence of nutrition. Another possible reason that we did not observe significant mediation is that metals such as arsenic and lead can directly affect immune function via epigenetic programming of leukocytes and altered metabolic physiology [88, 90–92]. Our results support the hypothesis that the earliest exposure periods represent the most susceptible developmental windows to immunotoxicity, as pregnancy exposures showed the strongest associations with antibody concentrations. All three metals have the potential to influence fetal development as they can readily cross the placenta [93–95]. Future work should focus on how nutritional factors during pregnancy may influence how certain metal mixtures modulate subsequent immune function.

Strengths of our study include the prospective design in a relatively large sample, with key exposure measurements taken during the critical developmental periods of pregnancy, toddlerhood, and early childhood. The measurement of vaccine-specific IgG antibodies provided us the opportunity to examine the long-term effects of metal exposures on relevant humoral immune outcomes. However, additional measurements of antibodies to other childhood vaccines could have provided additional clarity as to whether the associations are generalizable to other vaccines, such as live-attenuated or conjugate vaccines.

We utilized SEMs as a way to complement our MLR analyses and evaluate potential mediation. The SEMs allowed us to use a latent variable approach to examine the effect of a combined metal mixture on a combined antibody outcome, thus providing an additional method to address our hypothesis that metal mixtures are influencing humoral immunity. A primary strength of this analytical framework is that the information from several correlated exposure variables can be combined into a single analysis, which theoretically becomes more powerful than standard regression techniques [51]. The SEMs also improved issues surrounding multiple comparisons, as results obtained by MLR were not corrected for multiple comparisons. The SEM approach we presented did not explore potential interactions between metals. However, exploratory interaction models using MLR did not show significant metal-metal interactions in the overall population (data not shown). Future work in other study populations could benefit by further exploring potential interactions and nonlinear associations occurring in individuals with moderate to high-level exposures to all three of these metals.

Our study had a number of limitations. Drinking water was used to represent individual exposure to arsenic and manganese, which may have caused exposure misclassification. Exposures to arsenic and manganese among infants and mothers often occur via additional dietary sources outside of drinking water, so our reliance on this metric likely means we underestimated the true exposure levels. However, contaminated drinking water is the primary source of elevated exposures to arsenic and manganese in Bangladesh [84, 96]. Breastfeeding can reduce postnatal exposures, as arsenic and manganese are not fat soluble and typically not found in high concentrations in breast milk [47, 48]. Alternatively, infancy and childhood lead exposures can occur through diverse environmental sources, including breastmilk [38, 47, 59]. Therefore, we likely avoided major misclassification of environmental lead exposure by using B-Pb. There were differential metal-specific exposure profiles between our two primary study areas of Munshiganj and Pabna (higher relative W-As and B-Pb, respectively). While study area influenced the exposure distributions in this population, we do not consider it to have a direct causal relationship to vaccine-antibody concentrations. Adjusting or stratifying by factors hypothesized to only have an upstream association with exposures, but not outcomes, can induce analytical bias with unpredictable directionality [97]. Thus, clinic site was not included in our analyses as it does not pertain to potential biologically meaningful influences on the metal-antibody associations.

## Conclusion

Early life exposure to metals may modulate humoral immune function in childhood. Prenatal drinking water arsenic was negatively associated with diphtheria antibody, while blood lead was positively associated with tetanus antibody. However, drinking water manganese was not associated with antibody outcomes. Weight-for-age, a proxy of nutritional status, did not significantly mediate the indirect association between metal mixtures and antibody concentrations. Stronger associations were consistently observed among female children and during earlier windows of susceptibility. Our results provide evidence that exposure to multiple metals, especially during prenatal periods, may have diverse modulatory effects on humoral immunity among children.

## Supplementary information

**Additional file 1: Supplemental Material.**

## Data Availability

The datasets generated and analyzed during the current study are not publicly available due to privacy concerns, as they contain sensitive and protected health data on participants. Specific requests to access the data can be sent to molly.kile@oregonstate.edu.

## *Abbreviations*

B-Pb: Blood lead concentration
CFI: Comparative fit index
CI: Confidence interval
Ig: Immunoglobulin
LOD: Limit of detection
LRT: Likelihood ratio test
MICE: Multiple imputation in chained equations
MLR: Multiple linear regression model
PMM: Predictive mean matching
RMSEA: Root mean square error of approximation
SEM: Structural equation model
TLI: Tucker-Lewis Index
US EPA: United States Environmental Protection Agency
W-As: Drinking water arsenic concentration
WFA: Weight-for-age z-score
WHO: World Health Organization
W-Mn: Drinking water manganese concentration
