# Supplementary material for: Evaluating the effects between metal mixtures and serum vaccine antibody concentrations in children: a prospective birth cohort study
Source: Environ Health. 2020 Apr 10;19:41. doi: 10.1186/s12940-020-00592-z (PMC7146972; doi:10.1186/s12940-020-00592-z)
Supplement: Supplementary file 1 — Additional file 1: Supplemental Material. [file 12940_2020_592_MOESM1_ESM.docx]

Additional file 1: Supplemental Material

**Evaluating the effects between metal mixtures and serum vaccine antibody concentrations in children: a prospective birth cohort study.**

Barrett M Welch, Adam Branscum, G. John Geldhof, Sharia M. Ahmed, Perry Hystad, Ellen Smit, Sakila Afroz, Meghan Megowan, Mostofa Golam, Omar Sharif, Mohammad Rahman, Quazi Quamruzzaman, David C. Christiani, Molly L. Kile

| Table S1. Global model fit indices and criteria used to evaluate fit of structural equation models. | | | | |
| --- | --- | --- | --- | --- |
|  | Criteria for model fit | | | |
| Model fit index | Good | Acceptable | Poor | |
| Comparative fit index (CFI) | >0.95 | 0.90-0.95 | <0.90 | |
| Tucker-Lewis non-normed fit index (TLI) | >0.95 | 0.90-0.95 | <0.90 | |
| Root mean square error of approximation (RMSEA) | <.05 | 0.05-0.08 | >0.08 | |
| Note: All SEMs were required to meet good or acceptable fit for each model fit index. | | | |  |

Table S2. Results from adjusted multiple linear regression models restricted to girls (n=245).

|  | Arsenic | | Manganese | | Lead | |  |
| --- | --- | --- | --- | --- | --- | --- | --- |
|  | Change (%) (95% CI) | | Change (%) (95% CI) | | Change (%) (95% CI) | |  |
|  | Single element^a^ | Combined^b^ | Single element^a^ | Combined^b^ | Single element^a^ | Combined^b^ |  |
| Diphtheria |  |  |  |  |  |  |  |
| Pregnancy | -6.8 (-11.5, -1.7) | -5.8 (-11.2, -0.2) | -1.2 (-11.2, 10.0) | -0.5 (-10.8, 11.0) | 7.1 (-6.4, 22.6) | 3.4 (-10.2, 19.2) |  |
| Toddlerhood | 0.6 (-4.1, 5.5) | 0.9 (-3.9, 5.9) | 0.9 (-8.3, 11.1) | 0.3 (-9.1, 10.6) | 3.4 (-17.1, 28.9) | 2 (-18.4, 27.4) |  |
| Early childhood | 0.2 (-4.1, 4.8) | 0.3 (-4.1, 4.9) | 4.3 (-3.8, 13.1) | 3.7 (-4.4, 12.4) | 8.2 (-15.4, 38.5) | 2.7 (-20.6, 32.7) |  |
| Tetanus |  |  |  |  |  |  |  |
| Pregnancy | -4.6 (-9.7, 0.8) | -1.9 (-7.6, 4.1) | 7.8 (-3.6, 20.6) | 10.7 (-1.0, 23.8) | 24.5 (8.4, 42.9) | 23.6 (7.0, 42.9) |  |
| Toddlerhood | -2.7 (-7.5, 2.3) | -2.5 (-7.2, 2.5) | 0.2 (-9.5, 11.0) | -1.3 (-10.8, 9.3) | 3.9 (-18.1, 31.8) | 2.2 (-20.1, 30.7) |  |
| Early childhood | 0.2 (-4.4, 4.9) | 0.4 (-4.1, 5.1) | 3.2 (-5.2, 12.3) | 1.1 (-6.9, 9.9) | -1 (-23.4, 27.9) | -6.1 (-28.3, 23.0) |  |
| Note: Missing values of arsenic, manganese, and lead were estimated by multiple imputation. All models are adjusted for maternal education and breastfeeding duration. Outcomes are interpreted as percent change in median antibody concentration per doubling in given exposure.  ^a^ Models include includes single metal exposure category (arsenic, manganese, or lead) at all three periods of exposure.  ^b^ Model includes all metal exposure categories (arsenic, manganese, and lead) at all three periods of exposure. | | | | | | | |

Table S3. Results from adjusted multiple linear regression models restricted to boys (n=252).

|  | Arsenic | | Manganese | | | Lead | | |  |
| --- | --- | --- | --- | --- | --- | --- | --- | --- | --- |
|  | Change (%) (95% CI) | | Change (%) (95% CI) | | | Change (%) (95% CI) | | |  |
|  | Single element^a^ | Combined^b^ | | Single element^a^ | Combined^b^ | | Single element^a^ | Combined^b^ | |
| Diphtheria |  |  | |  |  | |  |  | |
| Pregnancy | -1.8 (-7.8, 4.6) | -1.5 (-8.0, 5.4) | | -1.7 (-10.5, 8.1) | -2 (-11.1, 8.1) | | 2.7 (-10.6, 17.9) | 5 (-9.5, 21.8) | |
| Toddlerhood | 1.6 (-4.1, 7.8) | 0.6 (-5.2, 6.9) | | 3.9 (-3.6, 11.9) | 3.9 (-3.7, 12.2) | | 5.6 (-16.5, 33.4) | 3.5 (-19.0, 32.4) | |
| Early childhood | 3.1 (-1.9, 8.3) | 3.9 (-1.4, 9.4) | | -4.6 (-11.3, 2.5) | -4.9 (-11.9, 2.6) | | -14 (-30.4, 6.2) | -9.9 (-27.6, 12.1) | |
| Tetanus |  |  | |  |  | |  |  | |
| Pregnancy | 4.1 (-2.8, 11.5) | 4.4 (-3.0, 12.4) | | 3.7 (-6.4, 14.8) | 2.5 (-7.8, 13.9) | | 0.4 (-13.7, 16.9) | 7.5 (-8.5, 26.3) | |
| Toddlerhood | 1.1 (-5.1, 7.7) | 0.8 (-5.5, 7.5) | | -4.2 (-11.6, 3.7) | -4.5 (-12.0, 3.7) | | -3.1 (-25.8, 26.6) | -0.4 (-23.9, 30.3) | |
| Early childhood | -0.6 (-5.8, 4.8) | 0.5 (-5.0, 6.3) | | -4.4 (-11.6, 3.3) | -4.1 (-11.7, 4.1) | | 3.9 (-17.7, 31.3) | 7 (-15.7, 35.7) | |
| Note: Missing values of arsenic, manganese, and lead were estimated by multiple imputation. All models are adjusted for maternal education and breastfeeding duration. Outcomes are interpreted as percent change in median antibody concentration per doubling in given exposure.  ^a^ Models include includes single metal exposure category (arsenic, manganese, or lead) at all three periods of exposure.  ^b^ Model includes all metal exposure categories (arsenic, manganese, and lead) at all three periods of exposure. | | | | | | | | |  |

## Results from non-imputed multiple linear regression models

Table S4. Non-imputed results from adjusted multiple linear regression models of diphtheria and tetanus antibody outcomes for single and combined metal exposures

|  | Arsenic | | Manganese | | Lead | |
| --- | --- | --- | --- | --- | --- | --- |
|  | Change (%) (95% CI) | | Change (%) (95% CI) | | Change (%) (95% CI) | |
|  | Single element^a^ | Combined^b^ | Single element^a^ | Combined^b^ | Single element^a^ | Combined^b^ |
| Sample size (n) | 469 | 322 | 469 | 322 | 322 | 322 |
| Diphtheria |  |  |  |  |  |  |
| Pregnancy | -3.3 (-7.3, 0.8) | -3.1 (-8.5, 2.6) | -0.4 (-7.3, 7.1) | -2.7 (-10.3, 5.6) | 7.4 (-4.4, 20.7) | 6.9 (-5.5, 20.8) |
| Toddlerhood | 1.6 (-2.2, 5.5) | 2 (-2.6, 6.8) | 2.9 (-3.0, 9.1) | 5.8 (-1.2, 13.2) | 9.4 (-7.6, 29.4) | 6.1 (-10.7, 26.0) |
| Early childhood | 1.2 (-2.2, 4.8) | 1.8 (-2.4, 6.1) | -1.4 (-6.8, 4.4) | -2.2 (-8.2, 4.1) | -11.1 (-26.4, 7.5) | -9.3 (-25.2, 10.1) |
| Tetanus |  |  |  |  |  |  |
| Pregnancy | 0.9 (-3.5, 5.5) | 3.3 (-2.8, 9.8) | 6.5 (-1.4, 15.1) | 2.5 (-6.1, 11.8) | 10.3 (-2.7, 25.0) | 13.3 (-0.7, 29.3) |
| Toddlerhood | -0.5 (-4.5, 3.6) | -0.9 (-5.7, 4.1) | -2.5 (-8.5, 3.8) | -1.7 (-8.6, 5.8) | 0.9 (-15.8, 20.9) | 3.2 (-14.2, 24.2) |
| Early childhood | -0.6 (-4.2, 3.2) | 0 (-4.4, 4.6) | -1.7 (-7.5, 4.5) | -4 (-10.3, 2.7) | 0.6 (-17.9, 23.3) | 2.6 (-16.7, 26.4) |
| Note: Missing exposure values were not imputed. All models are adjusted for maternal education, breastfeeding duration, and child sex. Outcomes are interpreted as percent change in median antibody concentration per doubling in given exposure.  ^a^ Models include includes single metal exposure category (arsenic, manganese, or lead) at all three periods of exposure.  ^b^ Model includes all metal exposure categories (arsenic, manganese, and lead) at all three periods of exposure. | | | | | | |

Table S5. Non-imputed results from adjusted multiple linear regression models restricted to girls (n=245).

|  | Arsenic | | Manganese | | Lead | |
| --- | --- | --- | --- | --- | --- | --- |
|  | Change (%) (95% CI) | | Change (%) (95% CI) | | Change (%) (95% CI) | |
|  | Single element^a^ | Combined^b^ | Single element^a^ | Combined^b^ | Single element^a^ | Combined^b^ |
| Sample size (n) | 233 | 156 | 233 | 156 | 156 | 156 |
| Diphtheria |  |  |  |  |  |  |
| Pregnancy | -6.3 (-11.3, -1.1) | -1.3 (-8.9, 6.8) | -0.3 (-10.6, 11.3) | -4.3 (-15.4, 8.4) | 16 (-2.0, 37.4) † | 15.3 (-3.7, 37.9) |
| Toddlerhood | 1.5 (-3.4, 6.7) | 1.9 (-4.3, 8.5) | 2 (-7.5, 12.5) | 7.4 (-4.9, 21.4) | 9.4 (-13.6, 38.4) | 8.1 (-15.3, 38.0) |
| Early childhood | -1.1 (-5.6, 3.6) | -1.5 (-7.2, 4.5) | 3 (-5.5, 12.3) | 1.3 (-8.3, 11.9) | -9.2 (-33.5, 23.9) | -13.3 (-37.5, 20.2) |
| Tetanus |  |  |  |  |  |  |
| Pregnancy | -3.9 (-9.2, 1.7) | 2.7 (-4.9, 10.9) | 8.7 (-3.0, 21.8) | 7.2 (-4.9, 21.0) | 28.3 (8.6, 51.5) | 29.3 (8.6, 54.0) |
| Toddlerhood | -2.2 (-7.2, 3.0) | -5 (-10.6, 1.0) | 2.8 (-7.1, 13.8) | 4.1 (-7.6, 17.1) | 1.7 (-19.3, 28.1) | 0.5 (-20.7, 27.5) |
| Early childhood | -0.8 (-5.6, 4.2) | -1.4 (-6.9, 4.5) | 0.3 (-8.4, 9.7) | -4.7 (-13.5, 5.0) | -2.6 (-28.2, 32.2) | -6.2 (-31.8, 28.8) |
| Note: Missing exposure values were not imputed. All models are adjusted for maternal education and breastfeeding duration. Outcomes are interpreted as percent change in median antibody concentration per doubling in given exposure.  ^a^ Models include includes single metal exposure category (arsenic, manganese, or lead) at all three periods of exposure.  ^b^ Model includes all metal exposure categories (arsenic, manganese, and lead) at all three periods of exposure. | | | | | | |

Table S6. Non-imputed results from adjusted multiple linear regression models restricted to boys (n=252).

|  | Arsenic | | Manganese | | Lead | |  |
| --- | --- | --- | --- | --- | --- | --- | --- |
|  | Change (%) (95% CI) | | Change (%) (95% CI) | | Change (%) (95% CI) | |  |
|  | Single element^a^ | Combined^b^ | Single element^a^ | Combined^b^ | Single element^a^ | Combined^b^ | |
| Sample size (n) | 236 | 166 | 236 | 166 | 166 | 166 | |
| Diphtheria |  |  |  |  |  |  | |
| Pregnancy | -2.4 (-8.4, 4.1) | -7.1 (-14.6, 1.1) | 0 (-9.3, 10.2) | -0.4 (-11.1, 11.6) | 4 (-11.7, 22.5) | 3 (-13.8, 22.9) | |
| Toddlerhood | 1.9 (-3.7, 7.9) | 1.9 (-4.9, 9.3) | 3.5 (-4.0, 11.6) | 6.8 (-2.0, 16.5) | 5.4 (-17.4, 34.4) | 0.1 (-22.1, 28.6) | |
| Early childhood | 3.6 (-1.5, 9.0) | 5.3 (-0.8, 11.7) | -4.8 (-11.8, 2.7) | -6 (-13.6, 2.3) | -10.9 (-30.1, 13.5) | -4.3 (-25.6, 23.1) | |
| Tetanus |  |  |  |  |  |  | |
| Pregnancy | 4.1 (-3.0, 11.7) | 1.3 (-7.9, 11.3) | 5.3 (-5.4, 17.1) | 0.4 (-11.7, 14.1) | 1.5 (-15.4, 21.9) | 7.4 (-12.1, 31.2) | |
| Toddlerhood | 1.2 (-4.9, 7.8) | 3 (-4.8, 11.4) | -5.2 (-12.7, 2.9) | -2.5 (-11.6, 7.5) | -5.5 (-28.0, 24.1) | -4.8 (-28.2, 26.4) | |
| Early childhood | -0.5 (-5.9, 5.1) | 1.5 (-5.0, 8.6) | -4.3 (-12.0, 4.0) | -4.2 (-12.9, 5.4) | 4.8 (-20.1, 37.3) | 9.2 (-17.8, 45.1) | |
| Note: Missing exposure values were not imputed. All models are adjusted for maternal education and breastfeeding duration. Outcomes are interpreted as percent change in median antibody concentration per doubling in given exposure.  ^a^ Models include includes single metal exposure category (arsenic, manganese, or lead) at all three periods of exposure.  ^b^ Model includes all metal exposure categories (arsenic, manganese, and lead) at all three periods of exposure. | | | | | | |  |
